# Human Tendon Stem/Progenitor Cell Features and Functionality Are Highly Influenced by *in vitro* Culture Conditions

**DOI:** 10.3389/fbioe.2021.711964

**Published:** 2021-09-20

**Authors:** Carlotta Perucca Orfei, Annie C Bowles, Dimitrios Kouroupis, Melissa A Willman, Enrico Ragni, Lee D Kaplan, Thomas M Best, Diego Correa, Laura de Girolamo

**Affiliations:** ^1^ Laboratorio di Biotecnologie Applicate all’Ortopedia, IRCCS Istituto Ortopedico Galeazzi, Milan, Italy; ^2^ Department of Orthopedics, UHealth Sports Medicine Institute, University of Miami, Miller School of Medicine, Miami, FL, United States; ^3^ Diabetes Research Institute and Cell Transplantation Center, University of Miami, Miller School of Medicine, Miami, FL, United States; ^4^ Department of Biomedical Engineering College of Engineering, University of Miami, Miami, FL, United States

**Keywords:** tendinopathy, Tendon Stem/Progenitor cells, culture density, inflammation, immunomodulation, substance P

## Abstract

Our understanding of tendon biology continues to evolve, thus leading to opportunities for developing novel, evidence-based effective therapies for the treatment of tendon disorders. Implementing the knowledge of tendon stem/progenitor cells (TSPCs) and assessing their potential in enhancing tendon repair could fill an important gap in this regard. We described different molecular and phenotypic profiles of TSPCs modulated by culture density, as well as their multipotency and secretory activities. Moreover, in the same experimental setting, we evaluated for different responses to inflammatory stimuli mediated by TNFα and IFNγ. We also preliminarily investigated their immunomodulatory activity and their role in regulating degradation of substance P. Our findings indicated that TSPCs cultured at low density (LD) exhibited cobblestone morphology and a reduced propensity to differentiate. A distinctive immunophenotypic profile was also observed with high secretory and promising immunomodulatory responses when primed with TNFα and IFNγ. In contrast, TSPCs cultured at high density (HD) showed a more elongated fibroblast-like morphology, a greater adipogenic differentiation potential, and a higher expression of tendon-related genes with respect to LD. Finally, HD TSPCs showed immunomodulatory potential when primed with TNFα and IFNγ, which was slightly lower than that shown by LD. A shift from low to high culture density during TSPC expansion demonstrated intermediate features confirming the cellular adaptability of TSPCs. Taken together, these experiments allowed us to identify relevant differences in TSPCs based on culture conditions. This ability of TSPCs to acquire distinguished morphology, phenotype, gene expression profile, and functional response advances our current understanding of tendons at a cellular level and suggests responsivity to cues in their *in situ* microenvironment.

## Introduction

Tendon injuries and pathologies are frequently painful and debilitating conditions affecting athletes and nonathletes alike. Historically, tendons are considered to be affected primarily by chronic degenerative events due to overuse. However, emerging research has revealed the presence of immune cells and inflammatory cytokines within tendons that might be key contributors to tendon-related disorders such as tendinopathies (Millar et al., 2009; Kendal et al., 2020). The role of inflammation that leads to a recruitment of immune cells to the site of the lesion is crucial. The interaction of these cells with tendon cells precedes establishment of an inflammatory amplification loop, which involves multiple alterations of the tissue matrix (Garcia-Melchor et al., 2021). Under physiological condition, the neural compartment is involved in normal movement of the body. However, it also plays an important role in the pathogenesis of tendinopathy as excessive stimulation leads to neo-innervation together with tissue breakdown and degeneration. The release of neuropeptides, such as substance P, stimulates the degranulation of mast cells, with the subsequent release of agents that modulate many cellular activities within the matrix (Scott and Bahr, 2009; Han et al., 2021; Millar et al., 2021). Despite the numerous advances in characterizing the pathogenesis of tendon disorders, a gold standard clinical treatment remains somewhat elusive (Millar et al., 2021). Basic research underpinning tendon biology and associated pathologies continues to be pursued to guide our development of novel evidence-based therapies.

New frontiers of tendon-related research are focusing on the study of a rare cell population harbored by the tissue exhibiting stem cell characteristics that represent an attractive and promising option for the development of more targeted treatments (Bi et al., 2007). Cells belonging to this heterogeneous population are termed tendon stem/progenitor cells (TSPCs) (Bi et al., 2007). They are generally defined as clonogenic, self-renewing and multipotent cells and expressing a surface antigen profile shared with mesenchymal stem cells (MSCs), for example, CD44+, CD90+, CD105+, CD146+, CD31-, and CD45- (Lui and Chan, 2011). TSPCs differ from MSCs for their transcriptional profiles with higher tendon-related gene expression levels (Tan et al., 2012). Many advances in the identification and characterization of distinct TSPC subpopulations have been made using single-cell analyses, giving a more complete view of the TSPC identity (Harvey et al., 2019; Mienaltowski et al., 2019; Kendal et al., 2020; Huang et al., 2021). TSPCs have attracted a lot of attention playing a key role in tendon development, homeostasis, and healing (Bi et al., 2007; Millar et al., 2021). However, several aspects concerning TSPCs are still controversial as to date no specific marker uniquely identifies these cells making their discrimination both *in vitro* and *in vivo* challenging (Lui, 2013). Overall, more efforts are needed to exploit their potential in a clinical setting. A growing number of studies have stated that a low-density culture method of isolated tendon cells would favor the growth of TSPCs (Rui et al., 2010; Mienaltowski et al., 2014; Lee et al., 2018; Wu et al., 2020) in culture. However, a relevant heterogeneity of methods is observed in characterization studies of TSPCs *in vitro* (Li et al., 2021). Starting from this premise, we wanted to assess the influence of culture condition on phenotypic and functional characteristics of TSPCs to further dissect their role in both physiological and pathological environment. In this regard, we performed modulation of density culture (Wu et al., 2020) by seeding TSPCs at both low (50 cells/ cm^2^) and high density (5000 cells/ cm^2^) and by performing a switch from low to high density during cell expansion to obtain a phenotype with hybrid features. The phenotypic, transcriptional, and secretory profiles of these three groups were characterized and comprehensively described. Furthermore, we investigated their gene expression and secretory activity responses to an inflammatory stimulus. We also explored both their immunomodulatory capacities and functions by co-culturing them with stimulated T cells and their ability to degrade the nociceptive stimulator substance P that is produced in the early phases of tendinopathy (Backman et al., 2011; Tran et al., 2020).

## Materials and Methods

### Study Approval

This study was performed at the University of Miami (UM-Miami) and IRCCS Istituto Ortopedico Galeazzi (IOG-Milan). Tendon tissue was obtained at the IOG from de-identified informed consented human donors prior to elective procedures for collection of waste materials. The protocol was approved by the local IOG Institutional Review Board (M-SPER-014-Ver.8-08.11.2016). After cell collections, samples were then transferred to UM-Miami where all other analyzes were performed. The study was conducted in accordance with the Declaration of Helsinki.

### Tendon Stem/Progenitor Cell Isolation and Cultures

**FIGURE 1:**
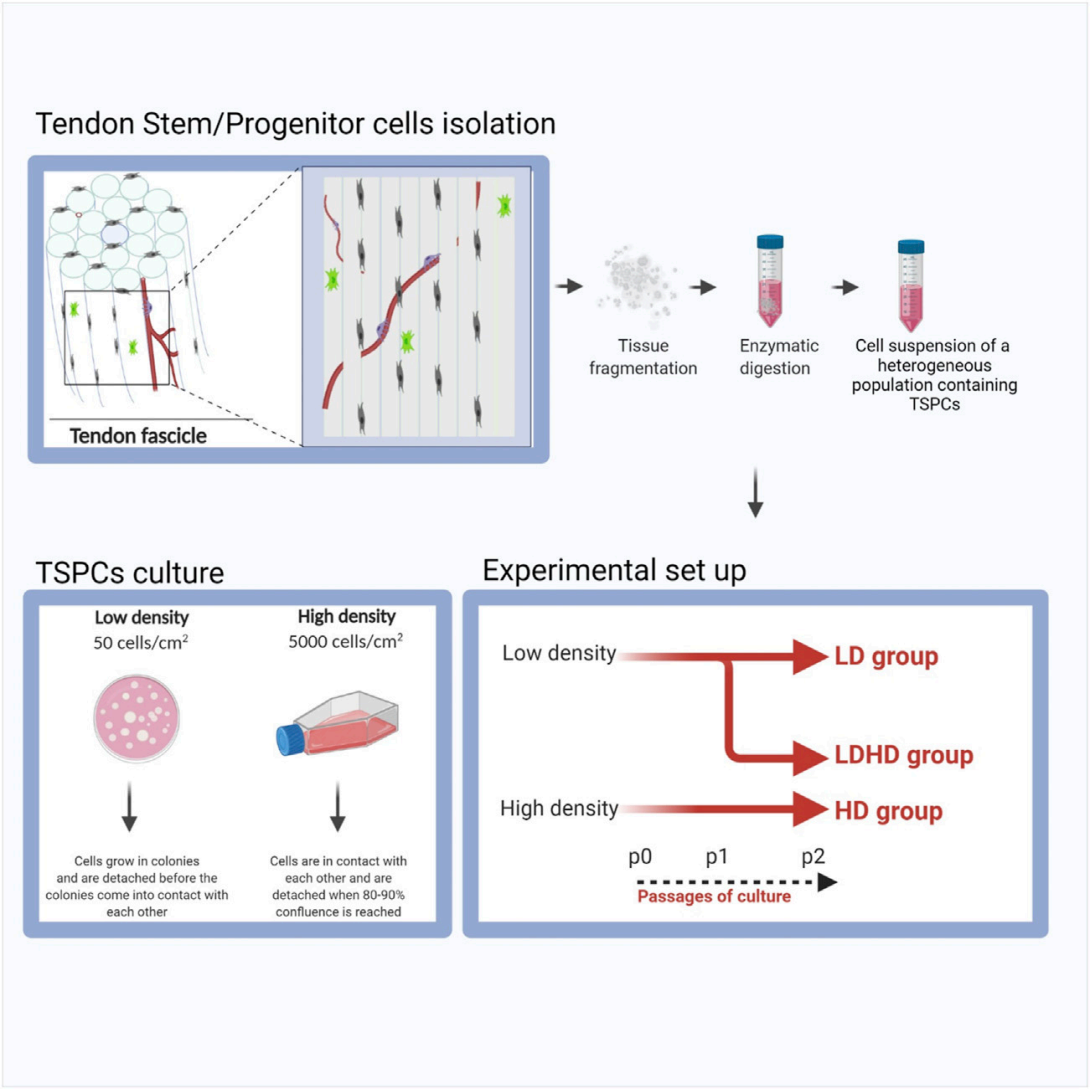
Schematic representation of the procedure used to isolate and culture LD, LDHD, and HD TSPCs.

Semitendinosus and gracilis tendons were collected from donors (n = 6, males, 33 ± 8 years/o) undergoing elective anterior cruciate ligament (ACL) reconstruction. Harvested samples were enzymatically digested with 0.3% w/v collagenase type I (185 U/mg, Worthington Biochemical Corporation) for 16 h to isolate human tendon stem/progenitor cells (TSPCs), cleaning the tendon from residues of other tissues. TSPCs were plated at low (50 cells/cm^2^, LD TSPCs) (Rui et al., 2010; Viganò et al., 2017; Wu et al., 2020) or high density (5000 cells/cm^2^, HD TSPCs) and cultured with low-glucose DMEM, L-glutamine, and penicillin-streptomycin (Life Technology), containing 20 and 10% FBS, respectively (GE Healthcare) at 37°C in 5% CO_2_. LD TSPCs grew as colonies. When colonies reached a particular size, LD was detached and reseeded in low density or in high density (the latter named as LDHD). The gauge of confluency was based on the percentage of colonies to remaining surface area where contact between the colonies was avoided. On the contrary, HD were detached at 80% of confluency and reseeded only in high density. Consequently, three groups were obtained thereafter (LD, HD, and LDHD). A schematic of the procedure used is represented in Figure 1. At passage 2, cells were evaluated for morphology, growth kinetics, and proliferation rate. Each cell culture condition was tested for growth kinetics by the IncuCyte® Live Cell Analysis System. Images were acquired at 10× magnification for morphology and analysis of the proliferation rate using IncuCyte ZOOM® software (Essen Bioscience).

### Cell Differentiation

Adipogenic, osteogenic, and chondrogenic differentiation assays were performed on LD, HD, and LDHD. For adipogenic differentiation, cells cultured with StemPro™ Adipogenic medium for 14 days (Gibco). For osteogenic differentiation, cells were cultured with StemPro™ Osteogenic Differentiation medium for 21 days (Gibco). All cells were fixed with 10% neutral buffered formalin (NBF) for 10 min, washed, and stained with respective stains: lipid droplet formation was detected by Oil Red O, whereas calcium deposition was detected by Alizarin Red. Images were acquired at 10× magnification, and stains were eluted for quantification. In brief, Oil Red O and Alizarin Red stained cells were incubated for 1 h in isopropyl alcohol or 10% cetylpyridinium chloride solution, respectively. Absorbance measurements of elusions were read using a plate reader at 584 nm optical density. De-stained cells were washed, and protein lysates were obtained using Pierce® RIPA Buffer (Thermo Fisher Scientific). Using a Pierce™ BCA Protein Assay Kit (Thermo Fisher Scientific), lysates were quantified for total protein. Adipogenesis and osteogenesis were represented as the OD values normalized to total protein for each sample.

Chondrogenic differentiation was induced on chondro-pellet cultures (0.25 × 10^6^ cells) by MesenCult-ACF differentiation medium (STEMCELL Technologies Inc). Sulfated glycosaminoglycans (sGAG) were quantified using the Blyscan Glycosaminoglycan Assay (Biocolor) according to manufacturer’s instructions after digestion (1 mg/ml papain solution overnight at 65°C). DNA was quantified using a Fluorescent DNA Quantitation Kit (Bio-Rad Laboratories). Histology for both hematoxylin and eosin (H&E) and 1% toluidine blue was performed on harvested and cryo-sectioned chondro-pellets. For each condition, samples not induced to differentiate served as controls.

### Phenotypic Analysis

Flow cytometric analysis was performed on LD, HD, and LDHD using a CytoFLEX flow cytometer (Beckman Coulter Life Sciences). 2 × 10^5^ cells were suspended in staining buffer and incubated for 20 min at 4°C with fluorescently conjugated anti-human antibodies: CD90-FITC (Clone 5E10, BioLegend), CD105-PE (Clone SN6h, BioLegend), CD44-BV605 (Clone IM7, BioLegend), CD73-APC (Clone AD2, BioLegend), CD166-PerCP-eFluor™ 710 (Clone 3A6, Fisher Scientific), CD14-APC (Clone 61D3, eBioscience), CD45-VioBlue (Clone 5B1, Miltenyi), CD31-PE (Clone WM59, BD Biosciences), HLA-DR-PE-CF594 (Clone G46-6, BD Biosciences), CD10-APC (Clone HI10a, Biolegend), CD146-PE (Clone 541-10B2, Miltenyi Biotec), CD200-FITC (Clone OX104, Invitrogen), CD133-PE (Clone TMP4, eBioscience), and CD107-PerCP/Cy5.5 (Clone H4A3, BioLegend). Acquisition of 50,000 events for each cell sample was performed. Subsequent gating strategies were standardized for each sample based on scatter, singlets, and positive expression, which were overlaid with corresponding isotype controls.

### Gene Expression

Total RNA from LD, HD, and LDHD was isolated with RNeasy® Plus Mini Kits (Qiagen) according to the manufacturer’s instructions. One microgram of RNA from each sample was then reverse-transcribed to obtain cDNA using a SuperScript™ VILO™ cDNA Synthesis Kit (Invitrogen). Following, 10 ng of cDNA of each sample, together with the QuantiFast SYBR Green qPCR kit (Qiagen) and primers selected for *PPAR*γ, *SOX9*, *RUNX2*, *SCX*, *TN-C*, *COL1A*, *COL3A1*, and β*ACTIN*, was used for the real-time quantitative polymerase chain reactions (qPCR) and analyzed with a StepOne Real-time thermocycler (Applied Biosystems). Human transcript primers were selected using PrimerQuest (Supplementary table 1).

### Inflammatory Priming

LD, HD, or LDHD designated for inflammatory induction was primed or not by incubation in culture media containing 10 ng/ml IFNγ and 15 ng/ml TNFα (R&D Systems) for 48 h, as previously performed (Kouroupis et al., 2019; Bowles et al., 2020). Cell supernatants were collected, centrifuged, and analyzed for the release of pro-inflammatory and anti-inflammatory factors. Cells were then detached and counted, and pellets were collected for gene expression analysis by using RT2 Profiler Arrays designed for Mesenchymal Stem Cell genes (Qiagen).

Master mixes containing 500 ng/ml of each cDNA sample, SYBR Green Supermix (Qiagen), and ultrapure water were prepared for each sample. Pathway-focused gene expression analysis was pre-assembled with primers of 84 genes related to known mesenchymal stem cells transcription profiles and five housekeeping genes (Supplementary table 2). Ct values were obtained and analyzed by the Qiagen’s Data Analysis Center. β*ACTIN* gene was selected after being tested with reference genes stability test (Ragni et al., 2019) as the optimal housekeeping gene and used to obtain the dCt values.

### Inflammation-Related Cytokines Production

Media collected of naive (non-stimulated) or primed cells were tested for production of inflammatory-related cytokines. Human C-Series ELISA Inflammation Arrays (RayBiotech Life, Inc.) were used. In brief, cell supernatants were collected, centrifuged, and transferred to new tubes to remove remaining cells and debris. Supernatants were incubated with each membrane according to the manufacturer’s instructions and imaged for densitometry measurements. Samples were normalized by background subtraction using the provided Excel-based analysis plug-in. All values were then normalized to cell counts, and data were expressed as densitometry values/total cells.

### Evaluation of the Immunomodulatory Capacities of Naive and Primed TSPCs

Prostaglandin E2 (PGE2) production was detected by single Elisa assay (Cayman Chemical). Moreover, the enzymatic activity of indolamine 2,3-dioxygenase (IDO), a cytoplasmic hemoprotein that oxidizes tryptophan yielding N-formylkynurenine (NFK), was evaluated on cell lysates by an IDO1 Activity Assay Kit (Abcam) according to manufacturer’s instruction. IDO metabolic activity was obtained by interpolating the fluorescence values obtained to the standard curve corresponding to the NFK concentration. The metabolic activity was then obtained as pmole of L-tryptophan metabolized by IDO during the reaction time. The level of expression of the indolamine 2,3-dioxygenase gene (primer sequence are reported in supplementary table 1) was evaluated by real-time PCR, as previously described.

### Immunopotency Assays With Stimulated T Cells

Naive or primed TSPCs (LD, HD, or LDHD) were co-cultured with T cells (n = 1) at a 1:2 ratio for 72 h to evaluate the TSPC immunomodulatory potential. Initially, human Pan T cells (STEMCELL™ Technologies) were thawed and cultured in flasks with complete RPMI containing 15% human serum AB (Corning), 1% 1 mM sodium pyruvate, 1% 0.1 mM nonessential amino acid, 1% 1X Vitamins, 1% 10 nM HEPES, and 1% 2 mM L-glutamine (Thermo Fisher Scientific). After culture recovery, T cells were stained with CellTrace™ CFSE Cell Proliferation Kit (Thermo Fisher Scientific) according to the manufacturer’s instructions and counted using live dead exclusion method. Simultaneously, complete RPMI replaced culture media in naive or primed LD, HD, and LDHD cohorts. Following, T cells were co-cultured directly with naive or primed TSPCs, at a 2:1 ratio (T Cells: TSPCs). ImmunoCult (STEMCELL™ Technologies) was used for T cell groups designated for stimulation and co-cultures were kept at 37°C in 5% CO_2_ for 72 h. After 72 h of co-culture, T cells were collected in each condition and stained with Ghost Dye™ Red 780 viability dye. Using Cytoflex LS (Beckman Coulter) and CytExpert software, at least 20,000 events were acquired for each sample. T cells were gated based on scatter, singlets, live/dead discrimination, and then positivity for CFSE. T cell proliferation rates (%) were calculated as [(CFSE^LOW^ events/ CFSE^+^) x100]. Remaining data were represented as the percent gated (%) of the reported phenotype.

### Substance P Degradation and CD10 Immunolocalization

Parameter substance P competitive immunoassay (R&D Systems) was used to quantify the levels of endogenous and exogenously added substance P (SP) to culture-expanded LD and HD (10^5^/well, 12-well; cells obtained from three donors) before and after priming, following manufacturer’s instructions and as previously described by our group (Kouroupis et al., 2019; Kouroupis et al., 2020). After 24 h in DMEM/10% FBS, cells were induced with inflammatory medium (supplemented with TNFα/IFNγ) for 48 h. SP was then quantified in centrifuged (1500 rpm; 5 min) conditioned media (in technical triplicates run in duplicates within the membrane) obtained from naive and primed TSPCs: 1) in baseline cultures (i.e., endogenous TSPC-derived SP); 2) after exogenous addition of substance P (720.6 pg/ml) for 35 min to the cell-free supernatant (i.e., supernatant group); and 3) after addition of SP (720.6 pg/ml) for 35 min to the cells in fresh medium (i.e., cells group). Parallel wells of supernatants and cells were treated with the CD10 inhibitor thiorphan (5 µg/ml) 30 min before and during SP addition. SP final levels were determined by subtracting measured optical densities of individual wells at 450 and 540 nm (SpectraMax M5 spectrophotometer), and converted into concentrations using the reference standard curve run with the assay, and contrasted to samples with only exogenously added SP to the medium (i.e., no cells and no supernatant).

Cells after fixation with 4% paraformaldehyde and washing with PBS were incubated with blocking solution (Tris-buffered saline–TBS containing 10% normal goat serum) for 1 h. Cells were incubated with 10 μg/ml goat anti-human CD10 polyclonal antibody (R&D System) in TBS with 1% normal goat serum for 1 h. Cells were then incubated with AlexaFluor594 conjugated rabbit anti-goat IgG secondary antibody at room temperature for 1 h. DAPI was used for nuclei staining. Microscope images were acquired with a Leica DMi8 microscope with Leica X software.

### Statistical Analysis

All the analyses were performed using GraphPad Prism v7.0 (Graphpad Software). Normality was assessed by Shapiro–Wilk tests. Statistical analyses between LD, HD, and LDHD were performed using non parametric one-way (ANOVA) by the Kruskal–Wallis test and Dunn’s multiple comparisons test. Comparisons between naive and treated cells were performed with the t-test by Wilcoxon matched pairs signed rank test. The level of significance was set at *p* < 0.05.

## Results

### Culture Density Influences TSPCs Morphology and Growth Kinetics

**FIGURE 2:**
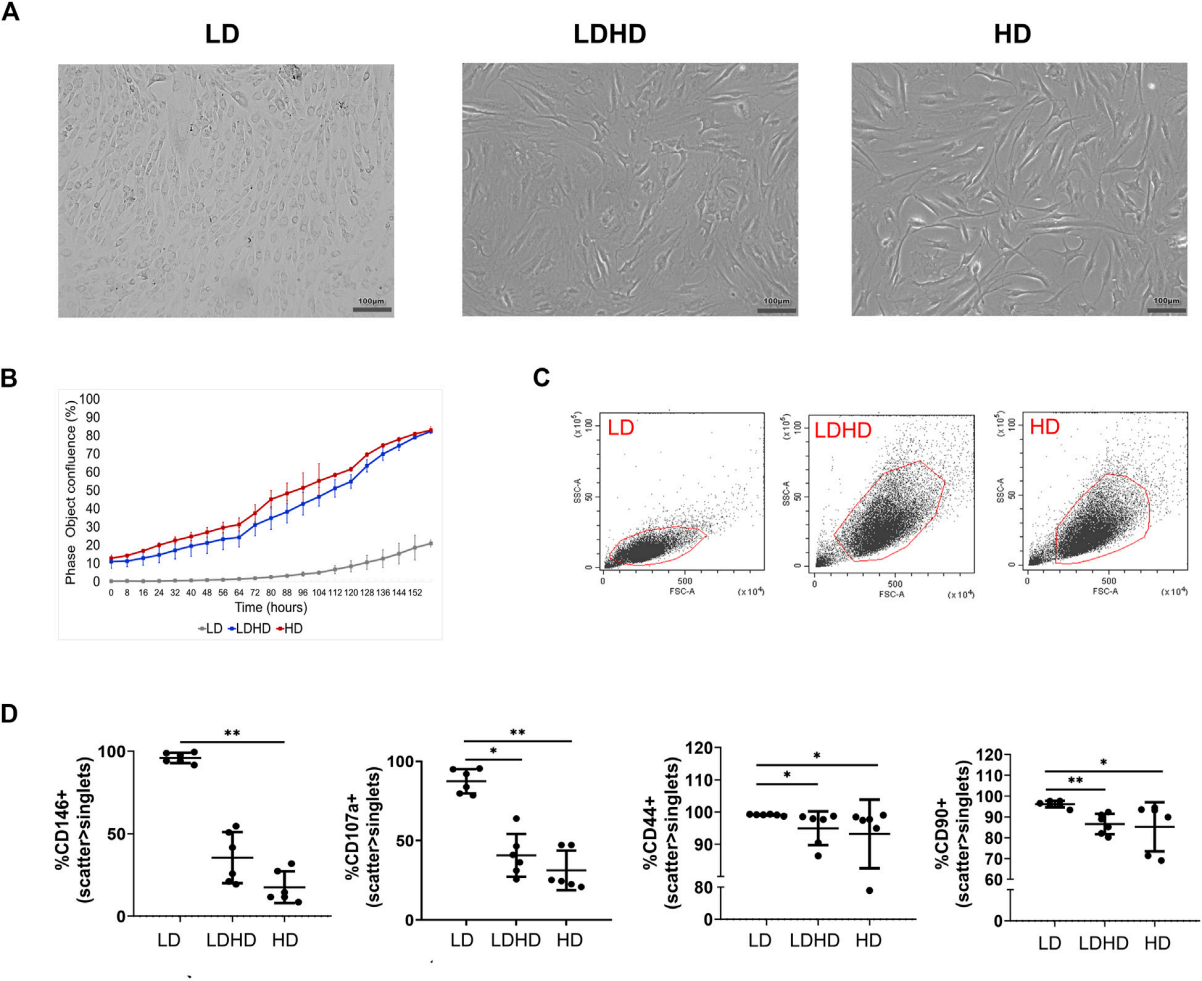
TSPCs phenotypes characterization (*n* = 6). **(A)** Representative bright-field images of LD culture showing a big colony of cobblestone like cells; LDHD and HD at 80% of confluency showing more elongated cells; scale bar: 100 µm. **(B)** Growth kinetics expresses as percentage of confluence. **(C)** Scatter parameters representation of each cell subset. **(D)** Percentages of positive cells for CD146, CD107a, CD44, and CD90 markers in LD, LDHD, and HD. Values are mean ± SD (*n* = 6). Differences among groups were tested using the Kruskal–Wallis test. *significantly different (*p* < 0.05), ** significantly different (*p* < 0.01). All the analyses were performed on cells at passage 2.

Culture of TSPCs at low density (50 cells/cm^2^, LD) up to passage 2 favored the growth of colony-forming cells exhibiting a cobblestone morphology (Figure 2). On the contrary, culture of cells at high density (5,000 cells/cm^2^, HD) that allows cell-to-cell contact, maintained a more pronounced fibroblast-like morphology (Figure 2A). The shift at passage 1 from low to high density resulted in a cell subset (LDHD) showing a fibroblast-like morphology similar to HD (Figure 2A). Different culture densities also resulted in different growth kinetic rates (Figure 2B). A two-way ANOVA was performed and evidenced that the interaction between the two variables (experimental group and time) is significant (*p* < 0.0001), highlighting a statistically different rate of proliferation among groups. Overall, statistically significant differences were observed between LD and LDHD group (*p* < 0.0001) and between LD and HD groups (*p* < 0.0001). No differences were observed between LDHD and HD groups.

### LD Are Phenotypically Different From LDHD and HD

The phenotypic analysis for MSC-defining markers was performed in all groups. The assessment of scatter parameters showed different cell size (FSC) and complexity (SSC) in LD compared to LDHD and HD (Figure 2C). A significantly enhanced expression of several markers was observed in LD when compared to LDHD and HD (Table 1 and Figure 2D). In detail, CD90, CD44, CD146, and CD107a markers were significantly higher expressed in LD than those in HD (Figure 2D). Interestingly, CD10, a known MSC marker with key functions described in other MSC types (Kouroupis et al., 2019; Kouroupis et al., 2020), was positively expressed by all groups at very high percentages (Table 1).

**TABLE 1:** Immunophenotypic analysis for commonly expressed MSC-defining markers.

	*LD*	*LDHD*	*HD*	*p values*
CD90	96.20 ± 1.57	86.63 ± 4.91**	85.27 ± 11.80*	0.0383 (LD vs HD)
				0.0074 (LD vs LDHD)
CD105	95.03 ± 7.24	95.17 ± 5.90	92.19 ± 13.98	n.s.
CD44	99.13 ± 0.23	94.97 ± 5.23*	93.22 ± 10.67*	0.0206 (LD vs LDHD)
				0.0148 (LD vs HD)
CD73	99.25 ± 0.15	90.63 ± 7.60	91.64 ± 9.14	n.s.
CD166	73.22 ± 21.19	48.88 ± 34.47	41.74 ± 31.03	n.s.
CD14	1.15 ± 0.68	0.13 ± 0.16	0.21 ± 0.14	n.s.
CD45	0.17 ± 0.07	0.45 ± 0.32	0.38 ± 0.43	n.s.
CD31	1.34 ± 0.76	2.0 ± 0.73	0.46 ± 0.37	n.s.
HLA-DR	1.02 ± 0.49	1.0 ± 0.71	0.44 ± 0.48	n.s.
CD133	1.17 ± 2.17	1.31 ± 1.72	1.35 ± 2.00	n.s.
CD146	96.01 ± 3.11	35.58 ± 15.50	17.62 ± 9.61 **	0.0011 (LD vs HD)
CD10	86.20 ± 9.36	87.95 ± 6.93	90.17 ± 5.41	n.s.
CD107a	87.43 ± 7.67	40.76 ± 13.50 *	31.29 ± 12.50 **	0.0385 (LD vs LDHD) 0.0024 (LD vs HD)

### LD, HD, and LDHD Exhibit Moderate Differences in the Multi-Differentiation Potential

**FIGURE 3:**
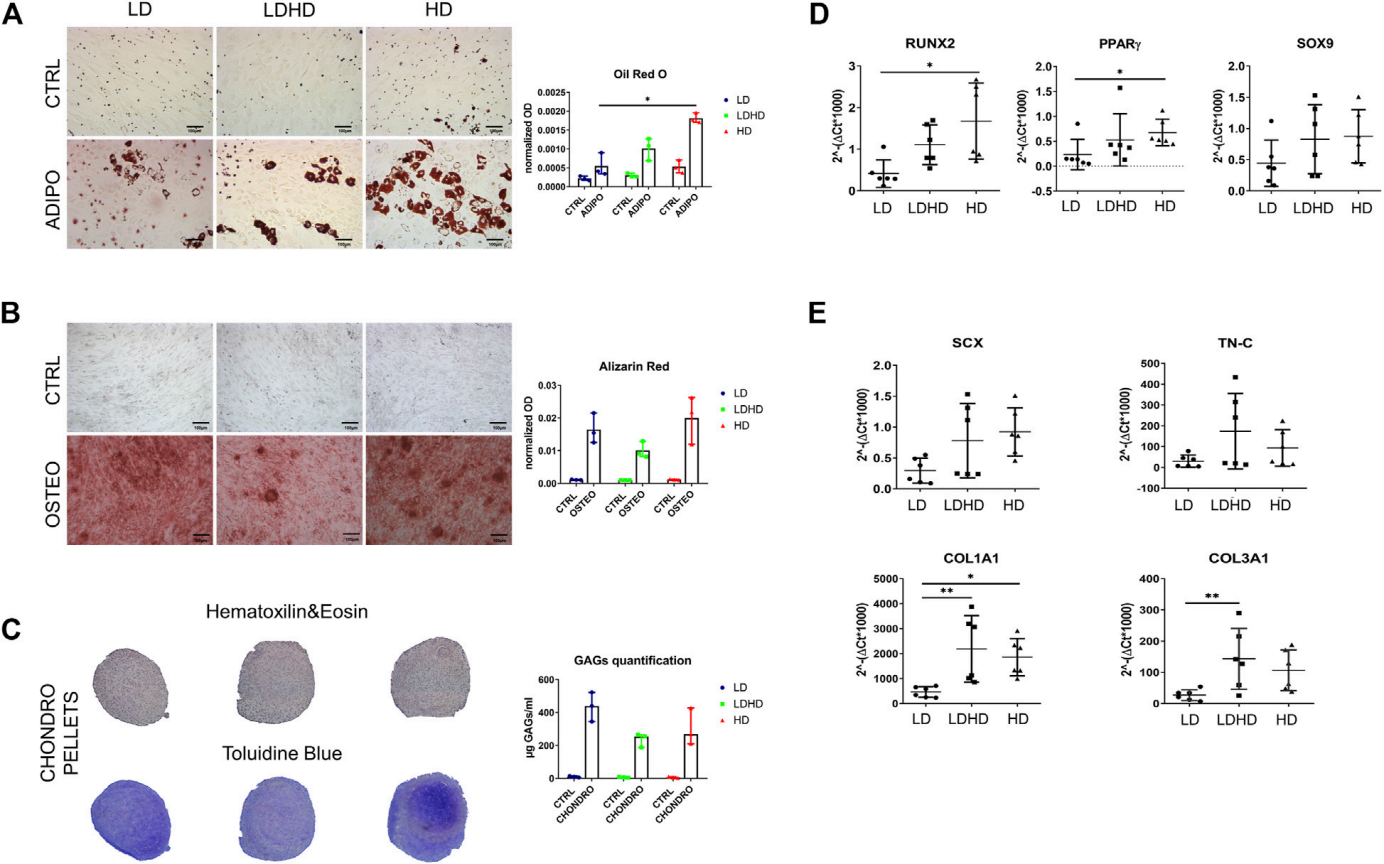
Multilineage differentiation capacity and gene expression profile. **(A)** Oil Red O and **(B)** Alizarin Red staining (scale bars: 100 µm) and quantification. Values are mean with range (*n* = 3). **(C)** Hematoxilin and eosin and toluidine blue staining of chondro-pellets and GAGs quantification after 21 days of differentiation. Values are mean with range (*n* = 3). **(D)** Transcription factors RUNX2, PPARγ, and SOX9 and **(E)** tendon-specific genes SCX, TN-C, COL1A1, and COL3A1 expression levels. Values are mean ± SD (*n* = 6). Differences among groups were tested using the Kruskal–Wallis test. *significantly different (*p* < 0.05), ** significantly different (*p* < 0.01).

Each group was assessed for adipogenic, chondrogenic, and osteogenic differentiation potential by standard differentiation protocols. HD showed a higher adipogenic differentiation compared to LD (*p* < 0.05), as revealed by quantification of Oil Red O staining for lipid droplets (Figure 3A). No significant differences were observed among groups for quantification of mineral deposition and glycosaminoglycans (GAGs) production which are markers of osteogenic and chondrogenic commitment, respectively (Figure 3B,C).

Key transcription factors *RUNX2* (osteogenesis) and *PPAR*γ (adipogenesis) were more expressed in HD than LD (*p* < 0.05), whereas the expression levels of the *SOX9* (chondrogenesis) gene showed a similar trend among all cell types without statistical significance (Figure 3D). In the same donors, the expression of tendon-related genes *SCX*, *TN-C*, *COL1A1*, and *COL3A1* was higher in LDHD than that in LD, with significant differences in expression levels of *COL1A1* and *COL3A1* genes only (*p* < 0.01). A significant difference was also observed between LD and HD in *COL1A1* expression (*p* < 0.05) (Figure 3E). No significant differences were observed in the expression levels of *TN-C* and *SCX* genes even though they were consistently lower in LD than in other groups.

### LD Exhibits Distinct Molecular Signatures With Higher Expression of LIF Gene

**FIGURE 4:**
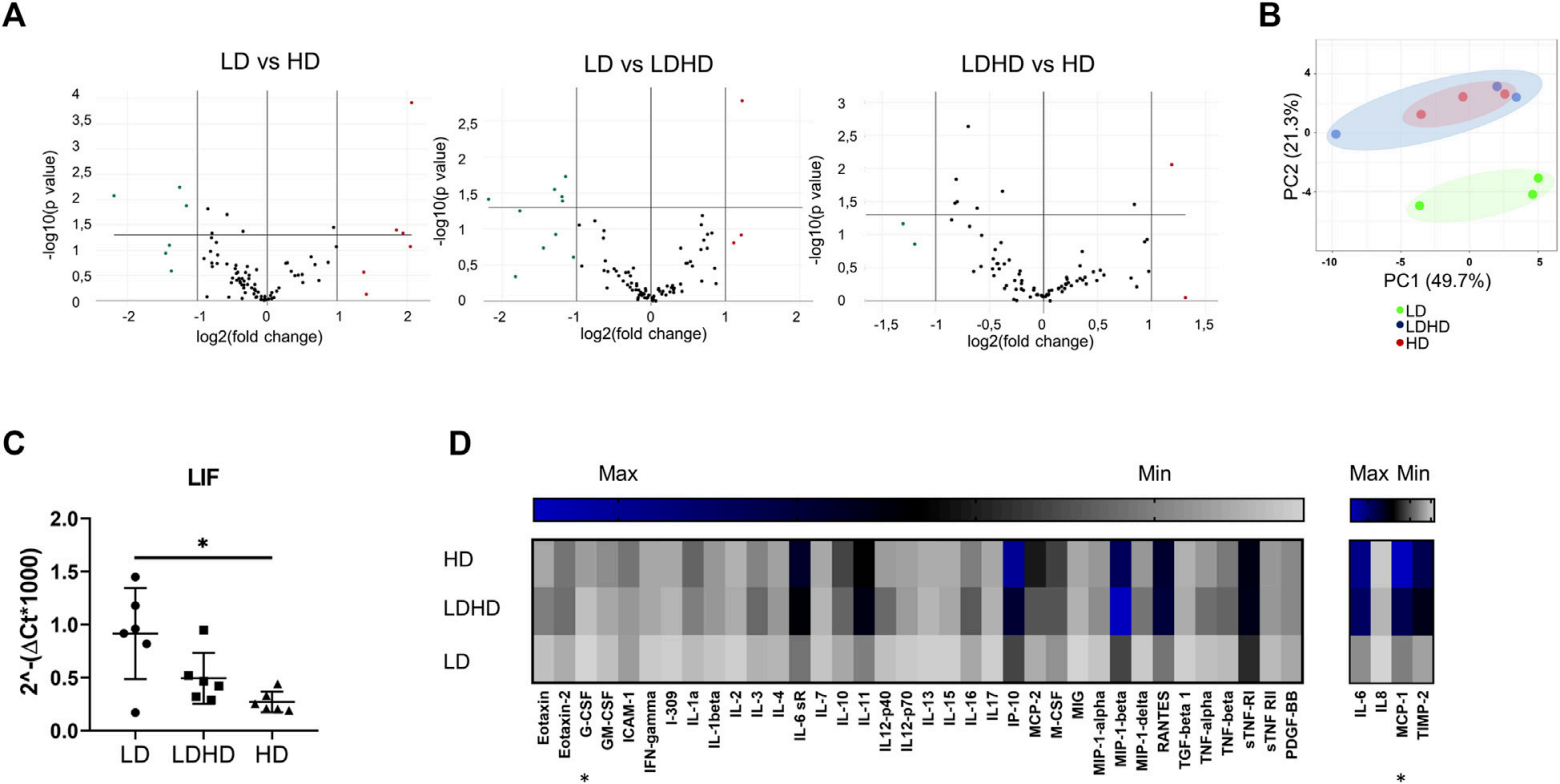
Transcriptional profiles and protein content release of naive TSPC groups. **(A)** Volcano plots representing over- (fold regulation >2, red-labeled) and under-expressed genes (fold regulation< 2, green labeled) of pair-wise comparisons (LD vs HD; LD vs LDHD; and LDHD vs HD) (*n* = 3). **(B)** Principal component analysis of dCt values of 84 MSC-defining genes in LD (green), LDHD (blue), and HD (red). Rows were centered and data were converted in ln (x + 1) values (*n* = 3). **(C)** LIF gene expression level in LD, HD, and LDHD. Values are mean ± SD (*n* = 6). Differences among groups were tested using the Kruskal–Wallis test. *significantly different (*p* < 0.05). **(D)** Heat map of inflammation-related proteins release. Pixel intensity was reported as arbitrary unit to semiquantitative protein production measurement of specific cytokines and chemokines. Values are mean (*n* = 3). Differences among groups were tested using the Kruskal–Wallis test. *significantly different (*p* < 0.05).

Profiling 84 MSC-defining genes revealed differences among the three groups after performing pair-wise comparisons (LD vs HD; LD vs LDHD; and LDHD vs HD) (Figure 4A). Over- and under-expressed genes are reported in the Table 2. Principal component analysis (PCA) plot was generated with ClustVis package (https://biit.cs.ut.ee/clustvis/) and calculated on dCt values by row centering and conversion of data in ln (x + 1) values. PCA revealed the overall clear transcriptional separation of LD from the other two groups (Figure 4B). The most striking differences were observed in the expression of leukemia inhibitory factor (LIF) gene, showing upregulation in LD, with a fold regulation of 4.19 (*p* < 0.001) and 2.34 (*p* < 0.05) when compared to HD and LDHD, respectively. Validation in all TSPC donors (n = 6) confirmed the presence of significantly higher expression of LIF in LD with the progressive decrease in LDHD and the lowest values in HD (*p* < 0.05) (Figure 4C).

**TABLE 2:** Over- and under-expressed genes in LD, LDHD, and HD.

Over-expressed genes			
	Gene symbol	Fold regulation	*p*-value
LD versus HD	BDNF	3.85	0.046434
	ICAM1	3.60	0.040022
	LIF	4.19	0.000121
LD versus LDHD	LIF	2.34	0.001652
LDHD versus HD	BDNF	2.28	0.008744
**Under-expressed genes**			
	**Gene symbol**	**Fold regulation**	***p***-**value**
LD versus HD	BMP4	−4.58	0.008376
	ITGA6	−2.38	0.005737
	SMURF2	−2.23	0.013229
LD versus LDHD	ENG	−2.22	0.018540
	FGF2	−2.29	0.035469
	IGF1	−4.54	0.038549
	ITGA6	−2.45	0.028135
	SMURF2	−2.28	0.040501

The basal secretion of inflammation-related mediators was measured for each group in naive conditions (absence of an inflammatory stimulation). A general “quiescent” condition was observed in LD compared to HD and LDHD (Figure 4D). Significant differences were observed only in the production of macrophage chemotactic protein-1 (MCP-1, *p* < 0.05 in LD vs HD), and granulocyte colony-stimulating factor (G-CSF, *p* < 0.05 in LD vs HD).

### The Inflammatory Insult Promotes Similar Molecular Responses in the Three Groups

**FIGURE 5:**
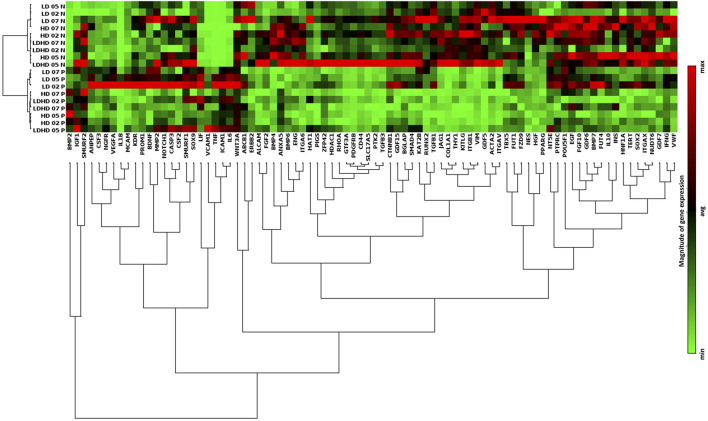
Transcriptional profiles and protein content release of primed TSPC subsets. **(A)** Hierarchical cluster diagram of genes expression profiles of both naive and primed cells of each group. Values are mean (*n* = 3). Data reported were elaborated by Qiagen–Gene Globe. The analysis was performed on cells at passage 2.

**FIGURE 6:**
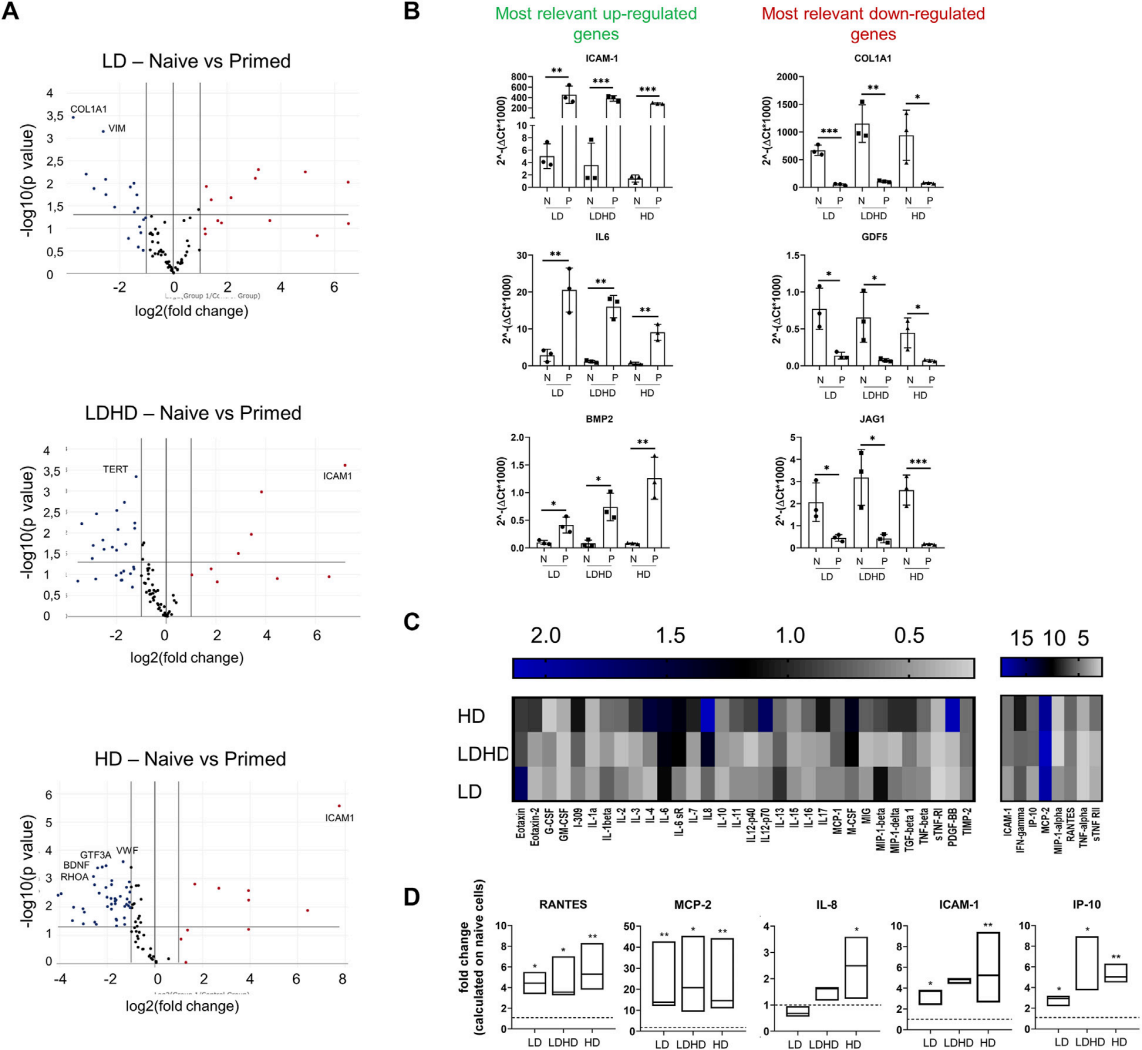
**(A)** Volcano plots representing over- (fold regulation >2, red-labeled) and under-expressed genes (fold regulation<2, blue labeled) of primed cells respect to naive ones for each TSPCs group (*n* = 3). The labeled genes represent those up- or downregulated with *p* < 0.001. Data are expressed as fold change [2^(−ΔΔCT)]. **(B)** Most relevantly up- (ICAM-1, IL-6, and BMP2) and downregulated (COL1A1, GDF-5, and JAG1) genes. Values are mean ± SD (*n* = 3). *p* values are calculated on Student’s t-test of the replicate 2^(−ΔCT) values for each gene in the naive and primed groups. *significantly different (*p* < 0.05), **significantly different (*p* < 0.01), ***significantly different (*p* < 0.001). Data reported in A) and B) were elaborated by Qiagen–Gene Globe. **(C)** Heat map of inflammation-related proteins release in cell supernatants. Data are reported as fold change of primed TSPC groups compared to naive ones. Pixel intensity was reported as arbitrary unit. Values are mean (*n* = 3). **(D)** Most relevantly modulated proteins levels RANTES, MCP-2, ICAM-1, IP10, and IL8 in cell supernatants. Data are reported as fold change of primed TSPCs groups compared to naive ones. Differences between naive and primed groups were calculated on unfolded data by multiple t tests.*significantly different (*p* < 0.05), **significantly different (*p* < 0.01).

The induction of an inflammatory microenvironment by the addition of TNFα and IFNγ to culture medium for 48 h (i.e., priming) resulted in an alteration of the transcription profiles for all groups. A clear distinction between naive and primed TSPCs was visible by cluster diagram analysis of gene expression values (Figure 5). Pair-wise comparison (naive vs primed) for each group showed various genes, among the 84 MSC-defining genes analyzed, with statistically significant differences in expression (Figure 6A). The complete list of genes and their respective *p* values are reported in Table 3. Specifically, the expression of *ICAM-1*, *IL-6*, and *BMP2* genes was significantly up-regulated (*p* < 0.05), while the expression of *COL1A1*, *GDF5*, and *JAG1* genes was significantly downregulated (*p* < 0.05) after priming in all groups tested (Figure 6B).

**TABLE 3:** Over- and under-expressed genes in primed cells *versus* naive.

Genes over-expressed in primed cells versus naive			
Cell type	Gene symbol	Fold regulation	*p*-value
LD TCs	BMP2	4.43	0.020900
	ICAM1	90.63	0.009442
	IL6	8.29	0.007743
	LIF	2.35	0.011781
	VCAM1	30.06	0.005588
	VEGFA	2.66	0.023215
LDHD TCs	BMP2	10.72	0.010867
	ICAM1	146.01	0.000243
	IL6	14.24	0.001051
HD TCs	BMP2	15.36	0.005698
	ICAM1	215.41	0.000003
	IL6	15.31	0.002610
	LIF	3.20	0.001551
	TNF	85.62	0.013239
**Genes under-expressed in primed cells versus naive**			
**Cell type**	**Gene symbol**	**Fold regulation**	**p-value**
LD TCs	ACTA2	−2.75	0.009980
	COL1A1	−13.14	0.000343
	GDF5	−5.67	0.017833
	GTF3A	−2.55	0.018051
	ITGAV	−7.70	0.013080
	ITGB1	−3.02	0.012064
	JAG1	−4.53	0.033778
	PDGFRB	−5.71	0.008198
	SLC17A5	−2.72	0.043550
	THY1	−2.49	0.035560
	VIM	−6.07	0.000705
LDHD TCs	BMP4	−5.84	0.014902
	COL1A1	−10.52	0.006057
	FZD9	−5.65	0.024838
	GDF5	−7.85	0.041174
	HGF	−3.99	0.026004
	ITGA6	−3.09	0.018789
	ITGAX	−2.41	0.007821
	ITGB1	−3.20	0.001871
	JAG1	−7.65	0.020030
	KITLG	−6.97	0.003515
	RUNX2	−3.96	0.002927
	TERT	−2.31	0.000452
	TGFB1	−2.40	0.005841
	THY1	−4.49	0.022009
	VIM	−3.83	0.008326
HD TCs	ACTA2	−3.10	0.026645
	ALCAM	−5.51	0.041017
	BDNF	−5.30	0.000415
	BGLAP	−2.74	0.004195
	BMP4	−5.80	0.001637
	BMP6	−3.59	0.004986
	BMP7	−2.05	0.010507
	CD44	−3.18	0.006170
	COL1A1	−11.04	0.030150
	ENG	−2.10	0.006763
	FGF10	−2.16	0.008957
	FUT1	−2.07	0.002947
	FZD9	−3.54	0.007883
	GDF5	−5.77	0.034187
	GDF6	−3.54	0.001617
	GDF7	−3.05	0.029016
	GTF3A	−4.14	0.000346
	HGF	−8.04	0.039234
	IFNG	−2.29	0.002009
	INS	−3.55	0.011871
	ITGAV	−8.11	0.011537
	ITGAX	−3.10	0.005613
	ITGB1	−3.98	0.009630
	JAG1	−15.35	0.003377
	KAT2B	−2.28	0.008039
	KITLG	−7.81	0.015201
	NUDT6	−3.19	0.005068
	PDGFRB	−10.67	0.010197
	PPARG	−3.06	0.046554
	PTPRC	−2.41	0.009465
	RHOA	−5.98	0.000833
	RUNX2	−4.03	0.004485
	SLC17A5	−3.12	0.005685
	SMAD4	−2.24	0.005194
	SOX2	−2.41	0.041429
	TERT	−2.43	0.024231
	TGFB1	−3.17	0.007642
	TGFB3	−3.51	0.001167
	THY1	−5.07	0.003230
	VIM	−4.64	0.000386
	VWF	−2.52	0.000250

### Priming Causes Similar Secretory Responses in LDHD and HD but Reveals a Stronger Immunomodulatory Aptitude of LD

The secretory profiles of primed TSPCs were compared to naive ones and represented as fold change values shown in the heat map (Figure 6C). Several cytokines and chemokines were significantly altered by the priming uniformly in all three groups. Among these, the secreted levels of regulated upon activation, normal T-cell expressed, and secreted protein (RANTES), monocyte chemoattractant protein-2 (MCP-2), ICAM-1, and interferon gamma induced protein 10 (IP-10) were significantly (*p* < 0.05) increased in primed cells of each cell group compared to naive (Figure 6D). No relevant differences were observed among primed LD, LDHD, and HD, suggesting similar secretory responsive profiles under inflammatory conditions. Differences were only observed in the secretion of interleukin 8 (IL-8) in the HD group, with a significant upregulation after priming that was not observed in the other group (*p* < 0.05).

Interactions among secreted molecules were evaluated by using the STRING database (https://string-db.org/) for functional protein association network establishment analysis. Pathway analyses after priming showed a consistent activation in all groups of four major signaling pathways: interleukin-17 (hsa04657, green-labeled), JAK-STAT (hsa04630, yellow-labeled), interleukin-10 (hsa-6783783, red-labeled), and interleukin−4 and −13 (hsa-6785807, blue-labeled) (Supplementary Figure 1).

**FIGURE 7:**
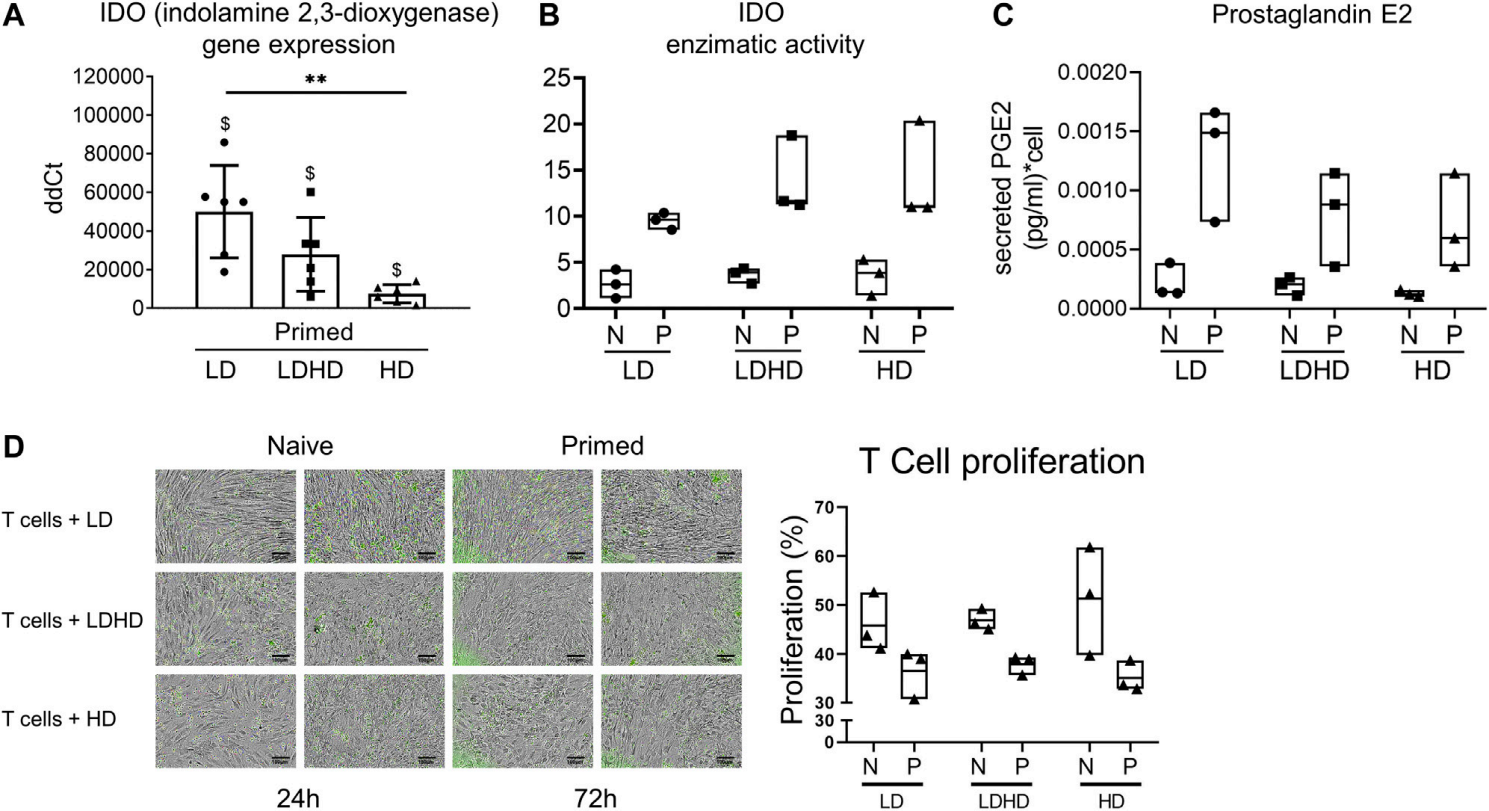
Immunomodulatory signature of TSPCs. **(A)** IDO gene expression level in LD, HD, and LDHD. Values are mean ± SD (*n* = 6). Differences among groups were tested using the Kruskal–Wallis test. **significantly different (*p* < 0.01). Differences between naive and primed groups were calculated on unfolded data by the Wilcoxon maTSPChed pairs signed rank test. ^$^ significantly different (*p* < 0.05). **(B)** IDO1 enzymatic activity. Data are expressed as µU/mg values and represented as median values with range (*n* = 3) **(C)** Secreted levels of Prostaglandin E2 in both naive and primed cells measured by ELISA Assay. Data are expressed as pg/ml of PGE2 per single cell and represented as median with range (*n* = 3). **(D)** Representative images of the stimulated T cells (green) co-cultured with both naive and primed LD, HD, and LDHD TSPCs at 24 h or 72 h of the 72-h duration of the IPA assay (*n* = 3). T-cell proliferation performed in the Immunopotency Assay co-culture with TSPCs showed relative immunosuppressive capacity of each TSPC group.

The role of the immunomodulatory enzyme indoleamine 2,3-dioxygenase (IDO) was evaluated in all groups before and after priming (Figures 7A,B). *IDO* gene expression was significantly upregulated in all groups after priming but more pronounced in LD (*vs* HD *p* < 0.05) (Figure 7A). IDO enzymatic activity was upregulated too in all samples after priming, however not reaching statistical significance (Figure 7B). Finally, the secretion of the immunomodulatory factor prostaglandin E2 (PGE2) showed a consistent increase in all primed compared to naive groups, although not statistically significant (Figure 7C).

### Immunopotency Assay Revealed Differences in T-cell Proliferation Between T cells Co-Cultured With Primed Versus Naive TSPC Cohorts

Quantitative comparisons of the % proliferation of stimulated T cells directly co-cultured with LD, LDHD, or HD TSPCs demonstrated the immunomodulatory effects of the corresponding TSPCs. Percentages of T-cell proliferation were comparable for T cells co-cultured with LD, LDHD, and HD with primed cohorts consistently suppressing T-cell proliferation more than naive cohorts. Although not statistically significant, Ppercent proliferation resulting from of stimulated T cells co-cultured with Naive LD (46 ± 6%), LDHD (47 ± 8%), or HD (51 ± 3%) was greater higher than the corresponding primed LD (37 ± 2%), LDHD (38 ± 2%), or HD (35 ± 7%) groups, revealing a stronger mitigation of T-cell proliferation by primed TSPCs (Figure 7D).

### Substance P Quantification and CD10 Immunolocalization Reveal the Ability of Both LD and HD to Degrade Substance P, Further Increased by Priming

**FIGURE 8:**
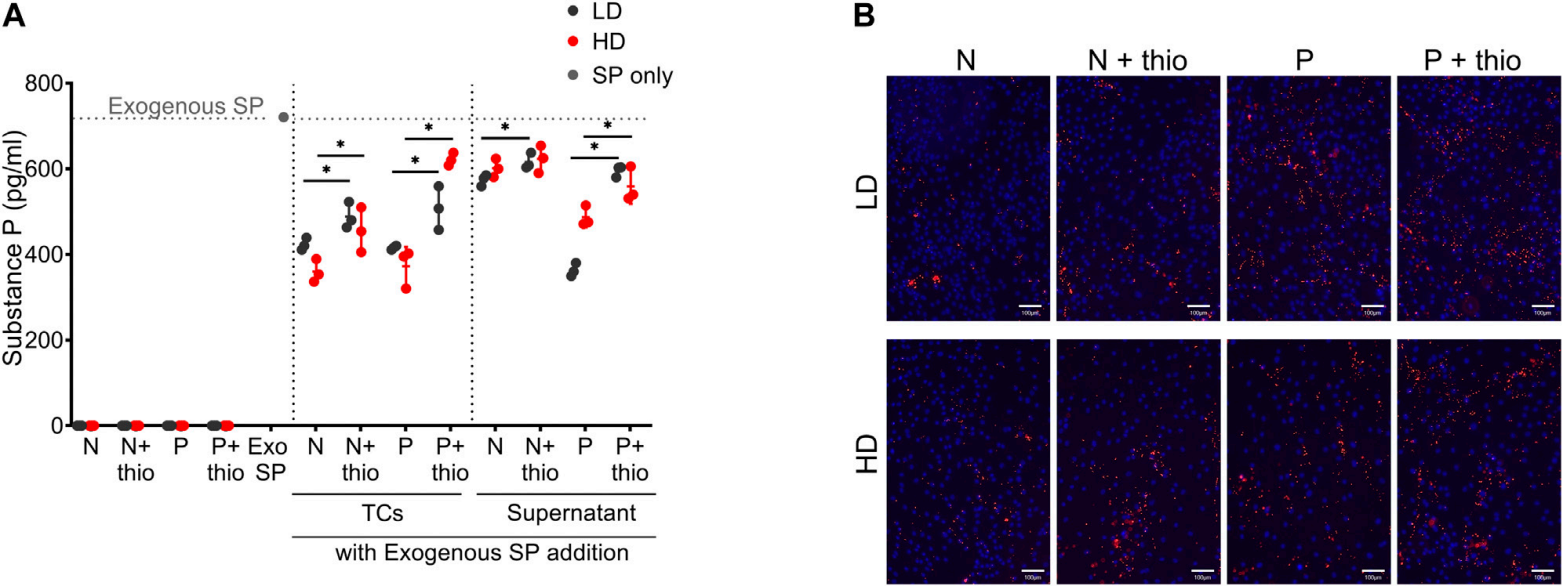
Substance P degradation and markers immunolocalization. **(A)** Quantification of endogenous and exogenously added substance P (SP) levels in LD and HD TSPCs, cells and supernatants, with or without CD10 inhibition with thiorphan (thio), in naive (N) or primed (P) conditions. Data are expressed as substance P concentration (pg/ml). Values are mean ± SD (*n* = 3). Differences among groups were tested using two-way ANOVA Tukey’s multiple comparisons test *significantly different (*p* < 0.05). **(B)** CD10 immunolocalization in naive (N) and primed (P) TSPCs LD and HD, with or without thiorphan addition (*n* = 3).

Endogenous SP was absent in both LD and HD with no changes after priming (Figure 8A). Importantly, the levels of exogenously added SP were significantly reduced (*p* < 0.05) by both LD and HD (naive and primed). Supernatants obtained from the same naive or primed TSPC cultures mirrored the SP degrading activities observed by cells, suggesting that the mode used by TSPCs to degrade SP is not only cell bound but also secreted in the culture supernatants.

In naive TSPCs, the increased CD10 expression, as indicated by phenotyping of both LD (86 ± 9%) and HD (90 ± 5%) cultures, was paralleled with the significant SP degradation. Thiorphan addition, an inhibitor of CD10 enzymatic activity, resulted in the significant abrogation of SP degradation (*p* < 0.05). Moreover, both LD and HD naive TSPCs showed a stronger SP degradation capacity compared to their supernatants, an effect that can be attributed to the increased presence of bound CD10 (concentrated punctuate signal on cell surface) on the TSPC cultures monolayer (Figure 8B). Upon priming, both LD and HD further increase their SP degradation capacity compared to naive cultures, an effect that is more evident in their supernatants. Again, these effects on SP were significantly abrogated by the addition of the CD10 inhibitor, thiorphan (*p* < 0.05) (Figure 8A). Interestingly, cell surface-bound CD10 was enriched in primed TSPCs compared to naive cultures (Figure 8B).

## Discussion

This study provides an in-depth characterization of human TSPCs, evaluating and comparing their phenotypes resulting from different culture conditions by analysis of morphology, growth kinetics, surface markers expression, multi-differentiation potential, gene expression profile, secretory activity, and immunomodulatory potential. We noted that human TSPCs demonstrate plasticity and have distinguishable responses to stimuli *in vitro*. First, dramatic differences among TSPCs were observed depending on the density culture they underwent, especially in terms of morphology, and immunophenotype and multi-differentiation potential. Moreover, LD, LDHD, and HD demonstrated to possess distinct *in vitro* functional responses to inflammatory stimuli as assessed by quantitative comparisons of naive and primed groups. Finally, preliminary observations on their immunomodulatory potential and their degrading ability of substance P suggest further distinct cellular responses. The culture at low density has been previously described and used in several studies to obtain the stem/progenitor cells of tendon *in vitro* (Bi et al., 2007; Rui et al., 2010; Ni et al., 2012; Viganò et al., 2017; Lee et al., 2018; Rui et al., 2019). In fact, avoiding cell-to-cell contact maintains the original self-renewal capacity and phenotype of MSCs (Balint et al., 2015). Our results are consistent with this ,observation and the LD TSPCs are distinguishable from LDHD and HD showing multiple features shared with MSCs.

LD, HD, and LDHD were evaluated for expression of tendon-related genes and for their multi-differentiation potential revealing striking differences. A significantly lower expression of *COL1A1* was observed in LD, both with respect to LDHD and HD. While not significant, similar results were found for the other tendon-related genes, suggesting that this particular group of cells may be less differentiated than the others. Moreover, lower gene expression levels of common multipotency genes (*RUNX2*, *PPAR*γ) were observed in LD. This set of observations, although not statistically robust, may suggest that this group is less likely to enter differentiation programs than other groups. However, a similar comparison performed on murine cells provided different results, showing a higher stem cell marker expression and earlier expression of tenogenic markers in tendon cells cultured at low density versus tendon cells cultured at high density (Lee et al., 2018). The conflicting results are not unusual as the disparities between human and murine MSC are well-documented (Ren et al., 2009; Su et al., 2014).

The immunophenotypic comparison of LD, HD, and LDHD also provided compelling differences in terms of the expression of stem cell defining markers (Dominici et al., 2006), especially CD90 and CD44, and the expression of CD107a that revealed higher levels in LD (87 ± 8%) than in LDHD (41 ± 13%) and HD (31 ± 12%). CD107a, also known as lysosomal associated membrane protein 1 (LAMP-1) has been previously described as a functional marker for the identification of natural killer cell activity (Alter et al., 2004) and more recently as a marker of immune cell activation and cytotoxic degranulation (Lorenzo-Herrero et al., 2019). To date, correlations of CD107a expression and function remain largely unknown in regards to MSCs, although we recently reported that a high expression of this marker in BMSCs can be attributed to an increased secretory activity, identifying them as “first responder” cells with therapeutically enhanced properties (Bowles et al., 2020). Phenotypic analysis for the CD146 surface marker revealed similar consistent differences with a markedly enhanced expression of LD compared to HD. CD146 was expressed in 96 ± 3% of LD contrasted to 18 ± 10% in HD and 36 ± 15% in LDHD. CD146 is a commonly used marker to identify pericytes (Tan et al., 2013; Schwartz et al., 2015) and those perivascular cells that constitute a fraction of the tendon stem/progenitor population (Lee et al., 2015). Recent studies have shown that a fraction of the tendon stem/progenitor niche is composed of pericytes (De Micheli et al., 2020) and that a cluster of tendon cells expressing high levels of CD90 and CD146 has been identified in the perivascular niche (Kendal et al., 2020).

Distinct molecular signatures were also observed with a notable difference in *LIF* gene expression that was constitutively higher in LD. LIF participates in both humoral and cellular immune responses (Patterson et al., 2002), with a key antagonistic effect on cytokines, through enrichment of regulatory T cells (Taga and Kishimoto, 1997; Gao et al., 2009). Moreover, LIF is involved in the differentiation process given its established role in the regulation of the embryonic stem cell self-renewal and maintenance of their pluripotency (Hirai et al., 2011).

The inflammatory priming promoted similar molecular responses in all TSPC groups, thus suggesting similar behaviors in such environments. Specifically, *ICAM-1* and *IL-*6 genes were consistently up-regulated in all groups. ICAM-1 generally influences T-cell activation and leukocyte recruitment to the site of inflammation, whereas IL-6 exerts pleiotropic roles in innate and adaptive immunity including participation in the inflammatory cascade, along with IL-8, through a defined IL-6/IL-8 ratio (Barajas-Gómez et al., 2017; Kouroupis et al., 2019; Kouroupis et al., 2020) and the maturation of B cells. Priming increased the expression levels of the *BMP2* gene, especially under the HD condition. This gene plays a critical role in the development of bone and cartilage by encoding a secreted ligand of the transforming growth factor-beta (TGF-β) superfamily and participation in the activation of SMAD-family transcription factor regulation (Wozney et al., 1988). BMP-2 promotes the deposition of GAGs and the expression of Aggrecan (*ACAN*) but decreased Decorin (*DCN*), Biglycan (*BGN*), and Fibromodulin (*FMOD*) expression levels in TSPCs (Rui et al., 2013). Therefore, an increase in the *BMP2* gene might affect the physiological structure of healthy tissue provoking a shift toward an undesirable microenvironment with altered extracellular matrix deposition (Dai et al., 2020). Clinical evidence supporting this hypothesis comes from the condition known as calcific tendinitis. The cause of this condition is still unclear but may be associated with a metaplastic transformation of tenocytes into chondrocytes that might induce calcification within the tendon (Chianca et al., 2018). In sharp contrast, priming elicited a consistent downregulation of *COL1A1*, *GDF5*, and *JAG1* in all groups. A mitigated expression of these genes may suggest dysregulation of tissue homeostasis. A decrease in *COL1A1* corresponds with a decrease in the production of Type I Collagen. Such reduction has been observed in a model of tenocyte and mast cell co-cultures (Behzad et al., 2013) which may confirm that inflammation and inflammatory cells exert a critical role in the development of tendinopathy. Downregulated *GDF5* may alter both homeostasis and tissue healing given its role in tendon development (Storm and Kingsley, 1999) and connective tissue formation (Wolfman et al., 1997). A downregulation of *JAG1* might alter cell physiology as this gene is strongly involved in the tenogenic and chondrogenic differentiation capacity of Sox9+/Scx + progenitor cells (Roberts et al., 2019). Dysregulation of this pathway has been implicated to favor chondrogenic differentiation (Roberts et al., 2019). Variability was observed in TSPC secretory profiles. The secreted levels of key regulatory molecules involved in immune-mediated and inflammatory processes including RANTES, MCP-2, ICAM-1 and IP-10 were significantly increased in all three groups after priming. In contrast, secreted interleukin-8 (IL-8) was significantly up-regulated in primed HD only, compared to naive HD. This cytokine is deeply involved in acute inflammation and acts as a potent chemoattractant and activator of neutrophils (Corre et al., 1999). Furthermore, its increase has been observed in tenocytes following stimulation with pro-inflammatory cytokines such as TNFα and IFNγ (Stolk et al., 2017).

Priming with pro-inflammatory cytokines is generally known to enhance the immunosuppressive effect of MSCs (Noronha et al., 2019). Their immunomodulatory effects have been attributed to the production of IDO and other immunomodulatory molecules such as PGE2, HGF, and TGFβ, following inflammatory signaling (Mellor and Munn, 2004; de Witte et al., 2015; Munn and Mellor, 2016). IDO acts on L-tryptophan catabolism, resulting in its reduction in the microenvironment with an increase in its metabolite, kynurenine, which exerts a crucial role on T cells and their activation, proliferation, and activity (Ankrum et al., 2014; Sinclair et al., 2018). Herein, a relevant upregulation of IDO expression and increased activity were observed in all three groups, with differences among them. Indeed, the upregulation of IDO was significantly higher in the LD than in HD, suggesting that the LD has a more pronounced immunomodulatory tendency. A similar trend, although not significant, was evident with the amount of secreted PGE2. Functional testing with an IPA assay suggested that the percent of T-cell proliferation was reduced in T cells/primed TSPCs co-cultures compared to naive ones, revealing comparable immunomodulatory effects by direct contact on T cells and modest differences among groups. Further exploration into more prominent immunomodulatory functions that are distinguishable among the groups may be warranted.

Regarding substance P (SP), naive LD, and HD exhibited a stronger cellular SP degradation capacity than their supernatants, an effect that was mitigated upon inflammatory priming. The functional capacity of the cells can be attributed to the expression of membrane-bound CD10 thus suggesting a CD10-dependent SP degradation mode of action. Priming induces upregulation and secretion of CD10, which was observed in the supernatant. This finding is in agreement with our previous reports indicating not only direct association of high CD10 cell levels with increased SP degradation but also with CD10 enzymatic activity via both membrane-bound and -released protein mechanisms (Kouroupis et al., 2019; Kouroupis et al., 2020). SP exerts a wide range of physiological effects with the most known roles relating to nociception and modulation of local neurogenic inflammatory and immune responses (Cao et al., 1998; Lehner et al., 2008; Mashaghi et al., 2016; Suvas, 2017; Zieglgänsberger, 2019). Moreover, regulation of SP activity is performed partly by cell membrane-bound neutral endopeptidase CD10/neprilysin (Maguer-Satta et al., 2011) that is expressed in multiple MSC types (Bühring et al., 2009; Bourin et al., 2013; Ong et al., 2014). Importantly, SP is a key molecule that mediates interactions between neurons and immune cells, with neuronal-derived SP affecting immune cell migration, proliferation, and cytokine production levels (Mashaghi et al., 2016). Additionally, studies have shown that SP is expressed in non-neuronal cell types such as macrophages (Mashaghi et al., 2016; Zieglgänsberger, 2019) that are the main immune cell infiltrate within the tendinopathy inflammatory microenvironment. Collectively, our results suggest that the cellular resident responders to inflammation may be involved in nociception via the secretion of a repertoire of molecules and specifically via the CD10/SP interaction.

The information gathered with this study helps understand better the role and functions of TSPCs and allows evaluating the way their properties are influenced by the culture conditions. This study, however, has some limitations that deserve consideration. For example, the different percentage of serum used for the culture of the three experimental groups can cause confusion. The LD TSPCs were supplemented with 20% FBS unlike the HDs, which received only 10% FBS. This difference was necessary because the newly isolated and low-density cultured cells would not have survived without such integration. However, further assessments should be made to confirm that any differences observed between groups were due to differences in FBS concentration.

In general, the uncertainty that still exists on the markers to be used to discriminate TSPCs from other cell types within tendon, such as tenocytes and tendon fibroblasts, which certainly represents a limitation in this field. This limitation is further aggravated in a broader sense by the lack of specificity of the minimum criteria for the identification of mesenchymal stem cells, which uniquely discriminate them from other cells (Soundararajan and Kannan, 2018). However, many advances are continually being made in the identification and characterization of distinct subpopulations of TSPCs, giving the evidence for a more complete view of their identity and functions.

## Conclusion

The results of this study improve our knowledge about the features of TSPCs *in vitro*, highlighting their robust plasticity in response to imposed external stimuli. Modulation of the cell density culture strongly influenced the identity of TSPCs *in vitro*, and this is of utmost importance for future cell characterization and functionality evaluation studies *in vitro*. To date, very few studies have investigated the spatial distribution of TSPCs *in vivo* (Tan et al., 2013). Therefore, a focus of future studies will be to understand to what degree our current findings can be extended to the *in vivo* environment. Our results confirm the heterogeneity of TSPCs and the presumed existence of multiple TSPC subpopulations, which may or may not be favored in culture based on the specific culturing conditions they are exposed to. Although preliminary, evidence reported and the differences found between the groups suggest that LD represents a key phenotype, to be explored and exploited for the development of targeted regenerative medicine therapies for tendon disorders.

## Data Availability

The original contributions presented in the study are publicly available. This data can be found here: https://osf.io/z3gbf/?view_only=1f9b50dfc9b64c2aaf1a61df5cfad808.
