# Supplementary material for: Human Tendon Stem/Progenitor Cell Features and Functionality Are Highly Influenced by in vitro Culture Conditions
Source: Front Bioeng Biotechnol. 2021 Sep 20;9:711964. doi: 10.3389/fbioe.2021.711964 (PMC8488466; doi:10.3389/fbioe.2021.711964)
Supplement: Supplementary file 1 [file DataSheet1.zip › Supplemental table 1.docx]

**Supplemental table 1: Human transcript primers**

| *PPARγ* | *FW:GCC TGC ATC TCC ACC TTA TT, RV:AGC GGG AAG GAC TTT ATG TAT G* |
| --- | --- |
| *SOX9* | *FW:GCA GGC GGA GGC AGA GGA G, RV:GGA GGA GGA GTG TGG CGA GTC* |
| *RUNX2* | *FW:ATA CCG AGT GAC TTT AGG GAT GC, RV:AGT GAG GGT GGA GGG AAG AAG* |
| *SCX* | *FW:AGA ACA CCC AGC CCA AAC, RV:CTG TCT TTC TGT CGC GGT C* |
| *TN-C* | *FW:CAC TAC ACA GCC AAG ATC CAG, RV:TCG TGT CTC CAT TCA GCA TTG* |
| *COL1A* | *FW:AGG ACA AGA AAC ACG TCT GG, RV:GGT GAT GTT CTG AGA GGC ATA G* |
| *COL3A1* | *FW:AAG TCA AGG AGA AAG TGG TCG, RV:CTC GTT CTC CAT TCT TAC CAG G* |
| *IDO* | *FW: AGA GTC AAA TCC CTC AGT CC, RV:AAA TCA GTG CCT CCA GTT CC* |
| *βACTIN* | *FW:AGA AAA TCT GGC ACC ACA C, RV:CTC CTT AAT GTC ACG CAC G* |
