# Supplementary material for: Human Tendon Stem/Progenitor Cell Features and Functionality Are Highly Influenced by in vitro Culture Conditions
Source: Front Bioeng Biotechnol. 2021 Sep 20;9:711964. doi: 10.3389/fbioe.2021.711964 (PMC8488466; doi:10.3389/fbioe.2021.711964)
Supplement: Supplementary file 1 [file DataSheet1.zip › Supplemental table 2.docx]

**Supplemental table 2: 84 genes related to known mesenchymal stem cells transcription profiles and 5 housekeeping genes**

| List of genes | | | |
| --- | --- | --- | --- |
| ABCB1 | ATP Binding Cassette Subfamily B Member 1 | JAG1 | Jagged Canonical Notch Ligand 1 |
| ACTA2 | Actin Alpha 2, Smooth Muscle | KAT2B | Lysine Acetyltransferase 2B |
| ALCAM | Activated Leukocyte Cell Adhesion Molecule | KDR | Kinase Insert Domain Receptor |
| ANPEP | Alanyl Aminopeptidase, Membrane | KITLG | KIT Ligand |
| ANXA5 | Annexin A5 | LIF | Leukemia inhibitory factor |
| BDNF | Brain Derived Neurotrophic Factor | MCAM | Melanoma Cell Adhesion Molecule |
| BGLAP | Bone Gamma-Carboxyglutamate Protein | MMP2 | Matrix Metallopeptidase 2 |
| BMP2 | Bone Morphogenetic Protein 2 | NES | Nestin |
| BMP4 | Bone Morphogenetic Protein 4 | NGFR | Nerve Growth Factor Receptor |
| BMP6 | Bone Morphogenetic Protein 6 | NOTCH1 | Notch Receptor 1 |
| BMP7 | Bone Morphogenetic Protein 7 | NT5E | 5'-Nucleotidase Ecto |
| CASP3 | Caspase 3 | NUDT6 | Nudix Hydrolase 6 |
| CD44 | CD44 | PDGFRB | Platelet Derived Growth Factor Receptor Beta |
| COL1A1 | Collagen Type I Alpha 1 Chain | PIGS | Phosphatidylinositol Glycan Anchor Biosynthesis Class S |
| CSF2 | Colony Stimulating Factor 2 | POU5F1 | POU Class 5 Homeobox 1 |
| CSF3 | Colony Stimulating Factor 3 | PPARG | Peroxisome Proliferator Activated Receptor Gamma |
| CTNNB1 | Catenin Beta 1 | PROM1 | Prominin 1 |
| EGF | Epidermal Growth Factor | PTK2 | Protein Tyrosine Kinase 2 |
| ENG | Endoglin | PTPRC | Protein Tyrosine Phosphatase Receptor Type C |
| ERBB2 | Erb-B2 Receptor Tyrosine Kinase 2 | RHOA | Ras Homolog Family Member A |
| FGF10 | Fibroblast Growth Factor 10 | RUNX2 | RUNX Family Transcription Factor 2 |
| FGF2 | Fibroblast Growth Factor 2 | SLC17A5 | Solute Carrier Family 17 Member 5 |
| FUT1 | Fucosyltransferase 1 (H Blood Group) | SMAD4 | SMAD Family Member 4 |
| FUT4 | Fucosyltransferase 4 | SMURF1 | SMAD Specific E3 Ubiquitin Protein Ligase 1 |
| FZD9 | Frizzled Class Receptor 9 | SMURF2 | SMAD Specific E3 Ubiquitin Protein Ligase 2 |
| GDF15 | Growth Differentiation Factor 15 | SOX2 | SRY-Box Transcription Factor 2 |
| GDF5 | Growth Differentiation Factor 5 | SOX9 | SRY-Box Transcription Factor 9 |
| GDF6 | Growth Differentiation Factor 6 | TBX5 | T-Box Transcription Factor 5 |
| GDF7 | Growth Differentiation Factor 7 | TERT | Telomerase Reverse Transcriptase |
| GTF3A | General Transcription Factor IIIA | TGFB1 | Transforming Growth Factor Beta 1 |
| HAT1 | Histone Acetyltransferase 1 | TGFB3 | Transforming Growth Factor Beta 3 |
| HDAC1 | Histone Deacetylase 1 | THY1 | Thy-1 Cell Surface Antigen |
| HGF | Hepatocyte Growth Factor | TNF | Tumor Necrosis Factor |
| HNF1A | HNF1 Homeobox A | VCAM1 | Vascular Cell Adhesion Molecule 1 |
| ICAM1 | Intercellular Adhesion Molecule 1 | VEGFA | Vascular Endothelial Growth Factor A |
| IFNG | Interferon Gamma | VIM | Vimentin |
| IGF1 | Insulin Like Growth Factor 1 | VWF | Von Willebrand Factor |
| IL10 | Interleukin 10 | WNT3A | Wnt Family Member 3A |
| IL1B | Interleukin 1 Beta | ZFP42 | ZFP42 Zinc Finger Protein |
| IL6 | Interleukin 6 | Housekeeping genes | |
| INS | Insulin | ACTB | Actin Beta |
| ITGA6 | Integrin Subunit Alpha 6 | B2M | Beta-2-Microglobulin |
| ITGAV | Integrin Subunit Alpha V | GAPDH | Glyceraldehyde-3-Phosphate Dehydrogenase |
| ITGAX | Integrin Subunit Alpha X | HPRT1 | Hypoxanthine Phosphoribosyltransferase 1 |
| ITGB1 | Integrin Subunit Beta 1 | RPLP0 | Ribosomal Protein Lateral Stalk Subunit P0 |
