# Supplementary figures and images for: Human Tendon Stem/Progenitor Cell Features and Functionality Are Highly Influenced by in vitro Culture Conditions
Source: Front Bioeng Biotechnol. 2021 Sep 20;9:711964. doi: 10.3389/fbioe.2021.711964 (PMC8488466; doi:10.3389/fbioe.2021.711964)

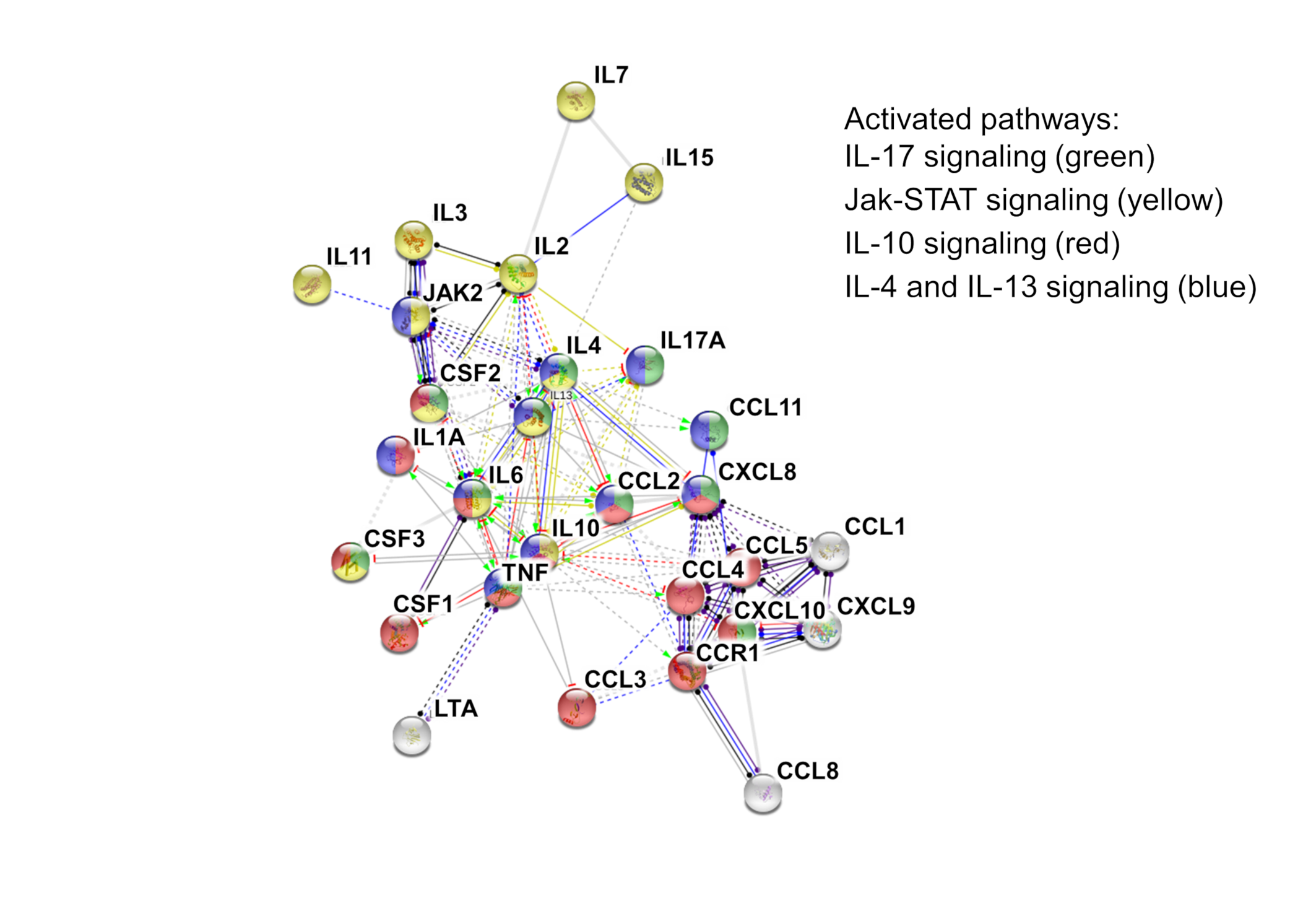

Supplement: Supplementary file 1 [file DataSheet1.zip › Supplemental figure 1.tiff]
